# A safety evaluation of profound hypothermia-induced suspended animation for delayed resuscitation at 90 or 120 min

**DOI:** 10.1186/s40779-017-0127-4

**Published:** 2017-05-30

**Authors:** Yu Liu, Shu Li, Zhi Li, Jian Zhang, Jin-song Han, Yong Zhang, Zong-tao Yin, Hui-shan Wang

**Affiliations:** 1 Department of Cardiovascular Surgery, General Hospital of Shenyang Military Command, Shenyang, 110016 China; 2 Department of Forensic Medicine, National Police University of China, Liaoning, 110035 China

**Keywords:** Profound hypothermia, Suspended animation, Resuscitation, Military combat casualty, Hemorrhagic shock

## Abstract

**Background:**

The successful treatment of military combat casualties with penetrating injuries is significantly dependent on the time needed to get the patient to an adequate treatment facility. Profound hypothermia-induced suspended animation for delayed resuscitation (SADR) is a novel approach for inducing cardiac arrest and buying additional time for such injuries. However, the time used to safely administer circulatory arrest (CA) is controversial. The goal of this study was to evaluate the safety of hypothermia-induced SADR over 90 and 120 min time intervals.

**Methods:**

Sixteen male BAMA minipigs were randomized into two groups: CA90 group (90 min, *n* = 8) and CA120 group (120 min, *n* = 8). Cannulation of the right common carotid arteries and internal jugular veins was performed to establish cardiopulmonary bypass for each animal. Through the perfusion of cold organ preservation solution (OPS), cardioplegia and profound hypothermia (15 °C) were induced. After CA, cardiopumonary bypass (CPB) was restarted, and the animals were gradually re-warmed and resuscitated. The animals were assisted with ventilators until spontaneous breathing was achieved. The index of hemodynamic perioperative serum chemistry values [alanine transaminase (ALT), aspartate aminotransferase (AST), creatinine (CR), lactic dehydrogenase (LDH) and troponin T (TnT)] and survival were observed from pre-operation to 7 days post-operation.

**Results:**

Fifteen animals were enrolled in the experiment, while 1 animal in CA120 group died from surgical error. All 8 animals in CA90 group recovered, with only 1 animal displaying mild disability. However, in CA120 group, only 2 animals survived with severe disability, and the other 5 animals died after 2 days post-operation. In CA90 group, the perioperative serum chemistry values increased at 1 day post-operation (ALT 84.43 ± 18.65 U/L; AST 88.99 ± 23.19 U/L; Cr 87.90 ± 24.49 μmol/L; LDH 1894.13 ± 322.26 U/L; TnT 0.849 ± 0.135 ng/ml) but decreased to normal or almost normal levels at 7 days post-operation (ALT 52.48 ± 9.04 U/L; AST 75.23 ± 21.46 U/L; Cr 82.69 ± 18.41 μmol/L; LDH 944.67 ± 834.32 U/L; TnT 0.336 ± 0.076 ng/ml).

**Conclusions:**

Profound hypothermia-induced SADR is an effective method for inducing cardiac arrest. Our results indicate that inducing CA for 90 min (at 15 °C) is safer than doing so for 120 min. Our results indicate that 120 min of CA at 15 °C is dangerous and can result in high mortality and severe neurological complications. Further experimentation is needed to determine whether 120 min of CA at temperatures lower than 15 °C can lead to safe recovery.

## Background

Military combat casualties resulting from penetrating injuries exsanguinate rapidly and can lead to cardiac arrest. Conventional resuscitation strategies for the treatment of exsanguinating traumatic cardiac arrest are poor. Furthermore, when trauma occurs in colder regions, the effect of treatment is lessened [1]. Although some traumatic injuries can be surgically treated [2], more than half of the trauma patients in recent military conflicts died because of limited medical care [3]. A previous study found that there were no survivors if the time before prehospital cardiopulmonary resuscitation (CPR) exceeded 10 min in blunt trauma victims or 15 min in penetrating trauma victims or if asystole occurred without pericardial tamponade [4]. Moreover, an additional study reported that 87.0% of 4596 casualties from 2001 to 2011 died before reaching surgical care, of which 75.5% (*n* = 3040) were classified as non-survivable (NS) and 24.3% (*n* = 976) as potentially survivable [5]. Thus, an effective and aggressive method to transport combat trauma patients, particularly potentially survivable patients, to medical treatment facilities could play a key role in improving survival rates.

Suspended animation for delayed resuscitation (SADR) is a novel approach for trauma patient resuscitation and provides increased time to apply surgical hemostasis and further treatment [6]. Suspended animation is the slowing or stopping of life processes by exogenous or endogenous means without termination. Therapeutic hypothermia has been traditionally used to protect the brain during cardiac surgery in order to finish repairs of complex congenital heart diseases and aortic arch surgeries [7, 8]. SADR is used to induce hypothermic preservation through cardiopulmonary bypass (CPB) during circulatory arrest in order to buy time for transport, damage control surgery, and delayed resuscitation. Thus, SADR is a particularly promising method for the effective treatment of potentially survivable patients and the improvement of their survival rates.

Preclinical studies based on SADR have been conducted in the last 3 decades using various animal models and have provided differing conclusions [9, 10]. Wu et al. [11] demonstrated that profound hypothermia (<10 °C) using glucose and oxygen in the flush solution can result in good recovery after 180 min of circulatory arrest (CA). However, others have only demonstrated good recovery after shorter CA time intervals. Rhee et al. [12] proposed that CA for 90 min with low flow or for 60 min with no flow, followed by delayed resuscitation, might obtain recovery in swine models. In contrast, in rat models, CA secondary to rapid exsanguination can be successfully treated by hypothermic flush in instances of CA that are less than 60 min, followed by delayed resuscitation [7, 9]. Nevertheless, high early mortality and multiple organ failure, including renal failure and liver necrosis, have been observed after resuscitation [13]. Rhee et al. [12] and Zink et al. [14] hypothesized that hypothermic arrest studies in the swine model might be most valuable for understanding treatment in humans because swine cardiopulmonary and immune physiology closely resembled those of humans. Additionally, the physiological response of swine models to hemorrhage resembles the human response more closely than any other non-primate species. Furthermore, the main ideas mentioned above were also reinforced by Alam et al. [15]. Here, we assessed survival and neurological outcomes in a swine model following complete cardiac arrest that was induced using organ preservation solution (OPS) in the blood and SADR at 15 °C with CA for 90 min and 120 min.

## Methods

### Animal preparation

The protocol for the study was approved by the Institutional Animal Care and Use Committee of General Hospital of Shenyang Military Command (2015019). All animals received humane care, and all animal protocols complied with the institution’s guidelines. Sixteen male BAMA minipigs, each weighing 41 to 76 kg (median weight: 49 kg and 25th–75th percentile ranges: 42–63 kg), were randomized into two groups: CA90 group (*n* = 8), which underwent 90 min of complete cardiac arrest, and CA120 group (*n* = 7), which underwent 120 min of complete cardiac arrest (1 swine died after terminal CPB caused by a blunt aortic injury from the femoral artery catheter). The animals were fasted overnight and sedated with an intramuscular injection of ketamine (10 mg/kg). After placement of an endotracheal tube, the animals were allowed to breathe spontaneously while light anesthesia was administered using isoflurane (0.5%–1.0%) through the Narkomed M anesthesia machine (North American Drager, Telford, PA). The right femoral arteries and veins were cannulated with 22G angiocatheters (SCW Medicath LTD, Shenzhen, GD) and 5F dual-lumen central venous catheters (SCW Medicath LTD, Shenzhen, GD), respectively. Puncture was used to monitor arterial pressure and central venous pressure. After the animals were heparinized (300 U/kg), the right carotid arteries and the internal jugular veins were cannulated with 16F femoral artery catheters and 18F femoral venous catheters (Edwards Lifesciences LLC, Irvine, CA), respectively. A cut-down technique was used to establish cardiopulmonary bypass. The animals were paralyzed and switched to full ventilatory support. Minimum ventilation was adjusted to keep the PaCO_2_ at 35–40 mmHg. The fraction of inspired oxygen (FiO_2_) was kept at the lowest possible level to maintain pulse oximetry readings above 95%.

### Hemorrhage protocol

Previous studies have shown that most mortalities (90.9%) in a cohort of casualties with potentially survivable wounds are associated with hemorrhage [5]. Therefore, we did not damage the vessels as in previous studies [15]. Rather, animals were bled via femoral venous catheters to approximate hemorrhaging [mean arterial pressure (MAP) rapidly decreases to 30 mmHg] and simulate traumatic laceration [16], and blood was collected in bags for auto-transfusion. All animals were kept in a state of hypoperfusion for 30 min (simulating transport time from the frontlines downrange to the military health center), with a 1000 to 1500 ml blood loss (approximately 50% of the estimated blood volume).

### CPB and induction of hypothermic arrest

**Fig. 1:**
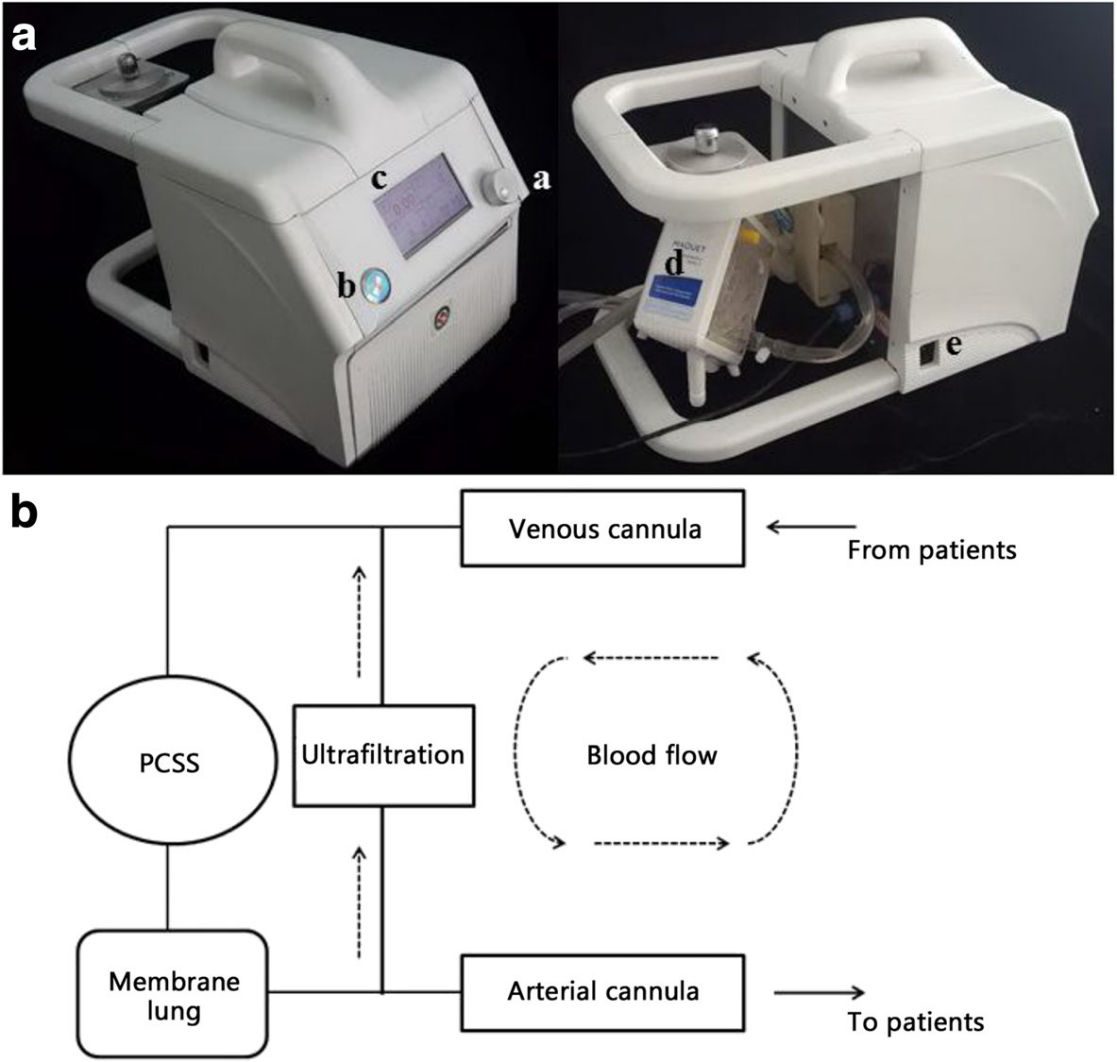
Portable cardiopulmonary support system (PCSS) and blood flow diagram. **a** Lateral view of the self-developed PCSS with the following components: *a*-flow adjust; *b*-switch; *c*-display screen; *d*-membrane Lung (Maquet); e-AC IN. **b** Diagram of the connection between the PCSS and the patient

Total body preservation was achieved using a Portable Cardiopulmonary Support System (PCSS) fitted with a centrifugal pump (self-developed, Fig. 1), a heat exchanger (Maquet Inc., Ann Arbor, MI) and ECMO kits (Maquet Inc., Ann Arbor, MI). The whole system was primed with 600 ml of the organ preservation solution (OPS), which was a simplified “Hypo Thermosol” solution designed by Taylor et al. [17] Two OPS solution treatments were used with either normal or high Table 1 (80.4 mmol/L) levels of potassium (OPS L and OPS H, respectively; ). Full cardiopulmonary bypass began at 3–4 L/min through the femoral artery catheters with 1.4 L of OPS H, which resulted in instantaneous cardioplegic arrest. Unlike previous studies [15, 16, 18], blood was not drained into a reservoir. The temperature of the heat exchanger was decreased as soon as possible. When the core temperature reached 20 °C, 2 L of blood was exchanged with 2 L of OPS L. Once the core temperature reached 15 °C, the flow was stopped, and suspended animation was induced. Blood was then drained through the venous tube by gravity and was collected in bags for auto-transfusion. After CA (90 or 120 min), the flow was increased to 3 to 4 L/min, and the temperature of the arterial return was adjusted to achieve desired re-warming rates (<0.5 °C/min) [19]. As the core temperature increased, all collected blood was gradually reintroduced to meet increasing oxygen demands. Electrolyte and acid-base abnormalities were also corrected as needed. Spontaneous cardiac activity typically resumed with the reversal of hyperkalemia and hypothermia. If required, internal cardioversion was performed, and mechanical ventilation was restarted. After a brief period of stabilization, the animals were gradually weaned off the CPB, and protamine sulfate was administered to reverse the heparin. All incisions were repaired, and the animals were extubated once normal respiratory drive had returned (typically 1–3 h). Intravenous injections of flurbiprofen axetil were administered for pain control, and Cefuroxime Sodium was administered at 24 h for perioperative antibiotic coverage. All animals were treated identically, except for the differences in CA time. Before the experiment, three animals were used to develop methodological details for experimentation. Fifteen animals without cannulation errors were included in the subsequent experiments.

**Table 1:** Content of organ preservation solution

Component	H Solution	L Solution
Ionic(mmol/L)		
Na^+^	102.4	134.2
K^+^	80.4	3.5
Ca^2+^	0.35	0.2
Mg^2+^	10.4	10.4
Cl^−^	84.2	126.2
SO_4_^2−^	10.4	10.4
pH buffers(mmol/L)		
HCO_3_^−^	15.0	20.0
Impermeants(mmol/L)		
Lactobionate	25.2	12.6
Mannitol	0	20.0
Glucose	10.5	10.5
Colloid(%)		
Gelatin	0.00	2.00

### Laboratory measurements and neurologic testing

Blood was drawn to measure liver enzymes (ALT, AST), renal function (creatinine), and markers of myocardial damage (LDH, TnT). Neurological status was assessed at 7 days post-operation using the overall performance category (OPC) index (1 = normal, 2 = mild disability, 3 = moderate disability, 4 = severe disability, 5 = death or brain death) [20].

### Statistical analysis

Normally distributed data are presented as group means ± SEM or SD, and non-normally distributed data are presented as the median and range of the distribution. Statistical analyses were conducted using SPSS version 22.0. Student’s *t* tests and Mann-Whitney *U* tests were used to compare continuous variables. Chi-square and Fisher’s exact tests were used to assess differences in the proportions of OPC values. Values of *P* < 0.05 were considered statistically significant.

## Results

### Hemodynamic and physiologic parameters

**Fig. 2:**
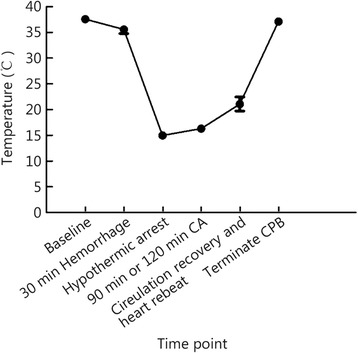
Changes in the core temperatures of test animals. Core temperatures during the experiment are shown for each time point from each animal in both groups

Hemorrhage by bleeding through femoral venous catheters caused a rapid decrease in MAP in the animals. The animals remained in profound shock during the 30 min of normothermic shock. An infusion of cold OPS H into the right carotid arteries resulted in the cessation of spontaneous cardiac activity, and during CA, there was no intrinsic cardiac output. At the end of the experiment, cardiac output returned to baseline in both groups. Changes in core body temperature are shown in Fig. 2. Upon the reversal of hyperkalemia and hypothermia, spontaneous cardiac activity returned. The results of physiologic and electrolyte values are shown in Table 2. There were no differences between the groups regarding the need for cardioversion or inotropic agents. Small doses of epinephrine were used (0.5 mg as an intravenous bolus for a maximum dose of 2 mg) for hypotension and bradycardia. Only lactate and glucose levels were lower in CA90 group compared with CA120 group at the off-CPB point, which could be due to the longer CA. The cooling and warming rates were the same in both groups.

**Table 2:** Physiologic, blood gas and electrolyte values (*x ± s*)

Time point	MAP (mm Hg)	BD (mmol/L)	Lactate (mmol/L)	Potassium (mmol/L)	Hematocrit (%)	Glucose (mmol/L)
Baseline						
CA90 group (*n* = 8)	90.88 ± 32.07	1.5 ± 2.7	2.77 ± 2.04	2.89 ± 0.49	30.13 ± 4.09	4.58 ± 1.93
CA120 group (*n* = 7)	99.43 ± 15.31	1.7 ± 5.4	4.05 ± 3.31	3.2 ± 0.57	32.14 ± 6.26	4.81 ± 2.17
Hemorrhage						
CA90 group (*n* = 8)	58.25 ± 9.77	0.5 ± 3.7	8.73 ± 2.57	2.77 ± 0.92	30.25 ± 2.78	5.96 ± 1.34
CA120 group (*n* = 7)	54.86 ± 14.23	0.4 ± 3.3	7.51 ± 1.82	2.96 ± 0.87	31.02 ± 4.81	6.1 ± 1.29
End of cooling						
CA90 group(*n* = 8)	-	1.1 ± 4.2	8.01 ± 1.22	9.36 ± 0.89	-	6.85 ± 1.30
CA120 group (*n* = 7)	-	−1.1 ± 4.1	8.24 ± 1.83	9.44 ± 1.88	-	7.19 ± 1.67
Beginning of warming						
CA90 group (*n* = 8)	-	−1.5 ± 5.7	9.48 ± 2.97	7.23 ± 1.05	-	7.56 ± 1.77
CA120 group (*n* = 7)	-	−1.1 ± 2.9	9.67 ± 1.45	7.14 ± 1.21	-	7.83 ± 1.31
Off CPB						
CA90 group (*n* = 8)	97.13 ± 25.51	1.1 ± 1.9	7.8 ± 1.25^(1)^	6.4 ± 1.07	25.88 ± 6.15	9.06 ± 1.65^(1)^
CA120 group (*n* = 7)	91.8 ± 14.82	0.4 ± 1.6	11.76 ± 3.18	5.81 ± 0.82	26.43 ± 5.65	11.3 ± 1.68

*MAP* Mean arterial pressure, *BD* Base deficit. (1) *P* < 0.05 compared with CA120 group. -: At end of cooling and beginning of warming, MAP were approximate 0 because of CA; Hematocrit were out of detection range of the blood gas analysis system

### Survival and neurologic outcomes

In CA90 group, all pigs survived to 72 h post-operation, and only 1 animal displayed paraplegia of the right legs, which may have been caused by ligation of the right femoral artery. In CA120 group, only 2 surviving animals exhibited paraplegia of their hind legs. Three CA120 pigs died at 48 h post-operation due to respiratory insufficiency. Two pigs also died intra-operatively with no spontaneous neurologic activity or reflexes. All animals in CA90 group achieved an OPC score of 1 by 7 days post-operation, and one animal achieved an OPC score of 2. In CA120 group, only 2 animals recovered to OPC 4, while another 5 animals were classified as OPC 5 (*P* < 0.05). These results are shown in Table 3.

**Table 3:** Over performance categories. Over performance categories (OPC 1–5) at 7 days after CA for 90 min or 120 min

Overall performance category	CA90 group	CA120 group
Death/brain death (OPC 5)		●●●●●
Severe disability (OPC 4)		●●
Moderate disability (OPC 3)		
Mild disability (OPC 2)	●	
Normal (OPC 1)	●●●●●●●	

Each dot represents one animal; *P* < 0.05, CA90 group vs. CA120 group

### Post-operative laboratory measurements

In CA90 group, biochemical abnormalities that were present during the post-operative period (ALT, AST, CR, LDH, TnT) were transient (Table 4). Abnormalities included an increase in the levels of liver aminotransferases, creatinine, lactate dehydrogenase, and TnT at 1 day post-operation. However, these levels decreased to normal or almost normal after 7 days post-operation. In CA120 group, there were no significant differences in biochemical indicators at the pre- and off-CPB time points. However, at 1 day post-operation, all biochemical indicators were higher in CA120 group than in CA90 group. Moreover, at 7 days post-operation, the increases in CA120 group relative to CA90 group were more pronounced, and there were only 2 surviving animals in CA120 group. Due to the low survival in CA120 group, we could not compare the differences between the 2 groups. Thus, only the raw data are shown (Table 4).

**Table 4:** Perioperative serum chemistry values

Time point	ALT (U/L)	AST(U/L)	CR (umol/L)	LDH(U/L)	TnT(ng/ml)
Pre-CPB					
CA90 group(*n* = 8)	35.95 ± 11.07	22.89 ± 10.88	77.46 ± 17.39	407.57 ± 109.02	0.009 ± 0.004
CA120 group(*n* = 7)	37.40 ± 9.50	23.64 ± 9.47	78.07 ± 27.99	459.67 ± 53.78	0.007 ± 0.003
Off-CPB					
CA90 group (*n* = 8)	34.94 ± 7.98	50.68 ± 21.55	65.61 ± 13.04	412.71 ± 159.07	0.132 ± 0.067
CA120 group (*n* = 7)	57.89 ± 21.75	46.12 ± 23.55	69.25 ± 22.12	416.67 ± 199.28	0.175 ± 0.075
Post-1d					
CA90 group (*n* = 8)	84.43 ± 18.65	88.99 ± 23.19	87.90 ± 24.49	1894.13 ± 322.26	0.849 ± 0.135
CA120 group(*n* = 5)	460.41 ± 164.71^(1)^	595.26 ± 148.26^(1)^	186.2 ± 39.37^(1)^	4943 ± 1277.96^(1)^	1.076 ± 0.208^(1)^
Post-7d					
CA90 group (*n* = 8)	52.48 ± 9.04	75.23 ± 21.46	82.69 ± 18.41	944.67 ± 834.32	0.336 ± 0.076
CA120 group (*n* = 2)	137.2; 208.1^(2)^	189.7; 217.4^(2)^	112; 161^(2)^	3125; 2708^(2)^	0.472; 0.512^(2)^

*ALT* Aspartate transaminase, *AST* Alanine aminotransferase, *CR* Creatinine, *LDH* Lactate dehydrogenase, *TnT* Troponin T, ^(1)^ *P* < 0.05 compared with CA90 group.^(2)^ is raw data

## Discussion

Trauma or hemorrhage that occurs in cold geographic regions can lead to increased mortality. One reason for this is trauma-induced accidental hypothermia, which may be a major cause of posttraumatic complications and adverse outcomes [21]. Therapeutic hypothermia can preserve the viability of key organs during lethal injury repair and can even be used to improve survival in the treatment of solid organ and bowel injuries [22]. SADR leverages the above mechanism to buy time for penetrating injury patients in military combat and would allow for the transfer of patients for further treatment while increasing the chances of neurologically intact survival [6]. However, several limitations in understanding the efficacy of SADR and its applicability in the field exist among previous studies. First, higher preservation times of at least 60 to 90 min are needed for medical evacuation and hemostasis [16]. Second, most SADR-related studies conduct OPS flushes without blood [7, 12, 16], which necessitates a large quantity of OPS for flushing and a large reservoir for collecting blood. Many cold liquids are not suitable for military combat transfer, and the choice of liquid is critical. Here, we used a total of only 4 L of OPS and a heat exchanger to induce SADR, which induced CA via OPS with blood. While the potassium concentration used in our experiment was very high (80.4 mmol/L), it was diluted by the blood in the animals. Previous studies have demonstrated that cardioplegia with blood positively influenced myocardial metabolism and reduced perioperative myocardial infarction [23, 24]. These studies and the results from our novel SADR approach may indicate that SADR induced with blood is particularly beneficial in terms of cardiac recovery.

The results reported here include several key points for the application of SADR in a combat setting. First, it is possible to induce SADR via CPB with PCSS in 20 to 30 min, which is in line with the amount of time necessary for hemostasis and evacuation. For instance, in a military combat setting, surgeons could immediately initiate hemostasis, cannulate the right carotid arteries and the internal jugular veins and connect to the PCSS for CPB to induce CA at the frontlines of combat. Profound hypothermia and SADR could be performed in a vehicle such as an ambulance, high speed railcar or helicopter. Second, OPS H containing 80.4 mmol/L K^+^ mixed with blood can induce cardioplegia at the K^+^ level of 20 mmol/L. After replacement by OPS L, K^+^ levels in our study decreased to 9 mmol/L. These K^+^ levels benefit the induction of cardioplegia and maintain the CA state, which is in accordance with previous studies [12]. Third, for CA90 group with no flow at 15 °C, organ viability was preserved, including that of the brain. All animals in CA90 group recovered well, except for one whose right leg exhibited paraplegia likely due to the ligation of its right femoral artery. Fourth, for CA120 group with no flow at 15 °C, most of the animals died, and the 2 surviving animals exhibited serious complications likely caused by brain or spinal cord injuries. However, a previous study [16] demonstrated that dogs can survive without brain injury after 120 min of CA at 10 °C. It is possible that the higher temperatures of CA used in the present study are responsible for the conflicting recovery results at 120 min. Fifth, a critical conclusion from this study is that blood must be drained through the venous tube by gravity after CA. Blood drainage can decrease the hydrostatic pressure in the vessel, which could aid in the avoidance of edema for important organs. In preliminary experiments, 2 out of 3 animals died from circulatory collapse. Autopsies revealed that the primary cause of death was pericardial tamponade caused by substantial hydropericardium. Additionally, seroperitoneum, hydrothorax, hepatomegaly and splenomegaly were all observed in the autopsies. It is possible that the edema-related side effects were caused by the lack of blood drainage. Finally, based on our results and those of previous studies, hypothermia is beneficial by reducing oxygen requirements and by other mechanisms, such as the reduction of excitotoxic apoptosis and inflammation [16]. Thus, more experiments are necessary to precisely identify the exact mechanisms by which hypothermia aids in SADR-induced recovery after traumatic injury.

The main limitation of this study is that there is a lack of histologic examination for organ systems, particularly for the brain. However, previous studies have suggested that microscopic brain changes do not lead to altered cognitive function [25]. Therefore, we chose to use the neurologic examination score to adequately reflect brain function. Regardless, histologic examination in concert with neurologic examination scores should be used in future studies. Additionally, a previous study [26] demonstrated that hemorrhage results in the rapid development of blood hypercoagulability. Hypercoagulability, in combination with low blood pressure and low cardiac output, can result in intravascular clotting that leads to tissue necrosis and infarction in some organs. However, pre-heparinization can prevent these hemorrhagic complications. Thus, future studies should compare hemorrhage without heparinization using our current methodology.

## Conclusions

We conclude that SADR can be effectively used in the recovery from trauma in organisms as induced by PCSS with OPS H and SADR at 15 °C for up to 90 min. However, our results also indicate that SADR at 15 °C for 120 min is dangerous and is not recommended for further trials. Thus, our results indicate that profound hypothermia-induced SADR is safer at 15 °C when conducted for 90 min rather than 120 min.

## Abbreviations

ALT: Aspartate transaminase
AST: Alanine aminotransferase
CA: Circulatory arrest
CPB: Cardiopulmonary bypass
CPR: Cardiopulmonary resuscitation
CR: Creatinine
LDH: Lactate dehydrogenase
MAP: Mean arterial pressure
OPC: Overall performance category
OPS: Organ preservation solution
PCSS: Portable cardiopulmonary support system
SADR: Suspended animation for delayed resuscitation
TnT: Troponin T

## References

[1] Wang HS, Han JS (2014). Research progress on combat trauma treatment in cold regions. Mil Med Res.

[2] Shoemaker WC, Peitzman AB, Bellamy R, Bellomo R, Bruttig SP, Capone A (1996). Resuscitation from severe hemorrhage. Crit Care Med.

[3] Bellamy R, Safar P, Tisherman SA, Basford R, Bruttig SP, Capone A (1996). Suspended animation for delayed resuscitation. Crit Care Med.

[4] Moore EE, Knudson MM, Burlew CC, Inaba K, Dicker RA, Biffl WL (2011). Defining the limits of resuscitative emergency department thoracotomy: a contemporary Western Trauma Association perspective. J Trauma.

[5] Eastridge BJ, Mabry RL, Seguin P, Cantrell J, Tops T, Uribe P (2012). Death on the battlefield (2001-2011): implications for the future of combat casualty care. J Trauma Acute Care Surg.

[6] Safar P, Tisherman SA, Behringer W, Capone A, Prueckner S, Radovsky A (2000). Suspended animation for delayed resuscitation from prolonged cardiac arrest that is unresuscitable by standard cardiopulmonary-cerebral resuscitation. Crit Care Med.

[7] Drabek T, Stezoski J, Garman RH, Han F, Henchir J, Tisherman SA (2007). Exsanguination cardiac arrest in rats treated by 60 min, but not 75 min, emergency preservation and delayed resuscitation is associated with intact outcome. Resuscitation.

[8] Seco M, Edelman JJ, Van Boxtel B, Forrest P, Byrom MJ, Wilson MK (2015). Neurologic injury and protection in adult cardiac and aortic surgery. J Cardiothorac Vasc Anesth.

[9] Drabek T, Stezoski J, Garman RH, Wu X, Tisherman SA, Stezoski SW (2007). Emergency preservation and delayed resuscitation allows normal recovery after exsanguination cardiac arrest in rats: a feasibility trial. Crit Care Med.

[10] Tisherman SA (2013). Salvage techniques in traumatic cardiac arrest: thoracotomy, extracorporeal life support, and therapeutic hypothermia. Curr Opin Crit Care.

[11] Wu X, Drabek T, Tisherman SA, Henchir J, Stezoski SW, Culver S (2008). Emergency preservation and resuscitation with profound hypothermia, oxygen, and glucose allows reliable neurological recovery after 3 h of cardiac arrest from rapid exsanguination in dogs. J Cereb Blood Flow Metab.

[12] Rhee P, Talon E, Eifert S, Anderson D, Stanton K, Koustova E (2000). Induced hypothermia during emergency department thoracotomy: an animal model. J Trauma.

[13] Keller ME, Aihara R, Lamorte WW, Hirsch EF (2003). Organ-specific changes in high-energy phosphates after hemorrhagic shock and resuscitation in the rat. J Am Coll Surg.

[14] Zink BJ, Stern SA, Mcbeth BD, Wang X, Mertz M (2006). Effects of ethanol on limited resuscitation in a model of traumatic brain injury and hemorrhagic shock. J Neurosurg.

[15] Alam HB, Chen Z, Ahuja N, Chen H, Conran R, Ayuste EC (2005). Profound hypothermia protects neurons and astrocytes, and preserves cognitive functions in a Swine model of lethal hemorrhage. J Surg Res.

[16] Behringer W, Safar P, Wu X, Kentner R, Radovsky A, Kochanek PM (2003). Survival without brain damage after clinical death of 60-120 mins in dogs using suspended animation by profound hypothermia. Crit Care Med.

[17] Taylor MJ, Bailes JE, Elrifai AM, Shih SR, Teeple E, Leavitt ML (1995). A new solution for life without blood. As an guineous low-flow perfusion of a whole-body perfusate during 3 hours of cardiac arrest and profound hypothermia. Circulation.

[18] Alam HB, Casas F, Chen Z, Smith WA, Reeves A, Velmahos G (2006). Development and testing of portable pump for the induction of profound hypothermia in a Swine model of lethal vascular injuries. J Trauma.

[19] Alam HB, Rhee P, Honma K, Chen H, Ayuste EC, Lin T (2006). Does the rate of rewarming from profound hypothermic arrest influence the outcome in a swine model of lethal hemorrhage?. J Trauma.

[20] Neumar RW, Bircher NG, Sim KM, Xiao F, Zadach KS, Radovsky A (1995). Epinephrine and sodium bicarbonate during CPR following asphyxial cardiac arrest in rats. Resuscitation.

[21] Hildebrand F, Radermacher P, Ruchholtz S, Huber-Lang M, Seekamp A, Flohe S (2014). Relevance of induced and accidental hypothermia after trauma-haemorrhage-what do we know from experimental models in pigs?. Intensive Care Med Exp.

[22] Sailhamer EA, Chen Z, Ahuja N, Velmahos GC, de Moya M, Rhee P (2007). Profound hypothermic cardiopulmonary bypass facilitates survival without a high complication rate in a swine model of complex vascular, splenic, and colon injuries. J Am Coll Surg.

[23] Zeng J, He W, Qu Z, Tang Y, Zhou Q, Zhang B (2014). Cold blood versus crystalloid cardioplegia for myocardial protection in adult cardiac surgery: a meta-analysis of randomized controlled studies. J Cardiothorac Vasc Anesth.

[24] Fang Y, Long C, Lou S, Guan Y, Fu Z (2015). Blood versus crystalloid cardioplegia for pediatric cardiac surgery: a meta-analysis. Perfusion.

[25] Connolly JE, Roy A, Guernsey JM, Stemmer EA (1965). Bloodless surgery by means of profound hypothermia and circulatory arrest. Effect on brain and heart. Ann Surg.

[26] Hardaway RM, Brune WH, Geever EF, Burns JW, Mock HP (1962). Studies on the role of intravascular coagulation in irreversible hemorrhagic shock. Ann Surg.

